# Sound Localization in Single-Sided Deaf Participants Provided With a Cochlear Implant

**DOI:** 10.3389/fpsyg.2021.753339

**Published:** 2021-10-21

**Authors:** Alexandra Annemarie Ludwig, Sylvia Meuret, Rolf-Dieter Battmer, Marc Schönwiesner, Michael Fuchs, Arne Ernst

**Affiliations:** ^1^Section of Phoniatrics and Audiology, Department of Otorhinolaryngology, University Hospital of Leipzig, Leipzig, Germany; ^2^Faculty of Life Sciences, University of Leipzig, Leipzig, Germany; ^3^Department of Otolaryngology, Unfallkrankenhaus Berlin, Berlin, Germany; ^4^Hearing Therapy Center Potsdam, Potsdam, Germany; ^5^Hospital of the University of Berlin, Charité Medical School, Berlin, Germany

**Keywords:** single-sided deafness, cochlear implant, sound localization, speech-in-noise, interaural time difference, interaural level difference

## Abstract

Spatial hearing is crucial in real life but deteriorates in participants with severe sensorineural hearing loss or single-sided deafness. This ability can potentially be improved with a unilateral cochlear implant (CI). The present study investigated measures of sound localization in participants with single-sided deafness provided with a CI. Sound localization was measured separately at eight loudspeaker positions (4°, 30°, 60°, and 90°) on the CI side and on the normal-hearing side. Low- and high-frequency noise bursts were used in the tests to investigate possible differences in the processing of interaural time and level differences. Data were compared to normal-hearing adults aged between 20 and 83. In addition, the benefit of the CI in speech understanding in noise was compared to the localization ability. Fifteen out of 18 participants were able to localize signals on the CI side and on the normal-hearing side, although performance was highly variable across participants. Three participants always pointed to the normal-hearing side, irrespective of the location of the signal. The comparison with control data showed that participants had particular difficulties localizing sounds at frontal locations and on the CI side. In contrast to most previous results, participants were able to localize low-frequency signals, although they localized high-frequency signals more accurately. Speech understanding in noise was better with the CI compared to testing without CI, but only at a position where the CI also improved sound localization. Our data suggest that a CI can, to a large extent, restore localization in participants with single-sided deafness. Difficulties may remain at frontal locations and on the CI side. However, speech understanding in noise improves when wearing the CI. The treatment with a CI in these participants might provide real-world benefits, such as improved orientation in traffic and speech understanding in difficult listening situations.

## Introduction

Orientation in the environment is a crucial ability in everyday situations, for instance in road traffic and communication in noisy surroundings. Acoustic information from both ears is necessary for locating a sound and understanding speech-in-noise accurately. Participants with normal hearing (NH) in one ear and deafness in the other ear (single-sided deafness, SSD) lack this binaural information. Approximately 200 new cases of SSD per million people are diagnosed each year (Baguley et al., 2006). While contralateral routing of sound devices (CROS hearing devices) and contralateral signal hearing through bone conduction (osseointegrated or bone-anchored hearing aids, BAHA®) are available, they do not restore hearing to the poor ear and therefore do not allow binaural hearing, because the brain only receives and processes auditory input from one side (Arndt et al., 2011). During the last years, cochlear implants (CIs) have been found to be useful to rehabilitate binaural hearing (van Hoesel and Tyler, 2003; Ching et al., 2004; Seeber et al., 2004; Dunn et al., 2008; Grossmann et al., 2016; Dillon et al., 2017a,b) and consequently enable sound localization in SSD participants (Firszt et al., 2012; Gartrell et al., 2014; Távora-Vieira et al., 2015; Grossmann et al., 2016; Litovsky et al., 2019; Wedekind et al., 2020).

Binaural input gives access to the main acoustic cues for horizontal sound localization, interaural time differences (ITD) for low-frequency sounds (below <1.5–2kHz), and interaural level differences (ILD) for high-frequency sounds (above >2–2.5kHz; Mills, 1958; Nordlund, 1962a,b; Rayleigh, 1907; Stevens and Newman, 1936; Yost and Dye, 1991; Wightman and Kistler, 1992; Recanzone et al., 1998; Carlile et al., 1999). Furthermore, it is known that lateralization of high-frequency sounds is also possible based on ITD (McFadden and Pasanen, 1976). There are several studies showing that this is achieved through processing of the envelope ITD (e.g., Bernstein and Trahiotis, 2014; Monaghan et al., 2015) probably with involvement of the lateral superior olive (Joris and Yin, 1995), a nucleus in which most of the neurons are tuned to high frequencies. Adults (Wightman and Kistler, 1992; Freigang et al., 2014, 2015) and children (Kühnle et al., 2013) with normal hearing can localize low-frequency noise better than high-frequency noise, because in these subjects, ITD cues contribute more than ILD cues to localization. The same is true for hearing-impaired children with bilateral sensorineural hearing loss provided with hearing aids (Meuret et al., 2018). Evidence suggests that bilateral cochlear implant users rely mostly on ILD in quiet listening situations (van Hoesel and Tyler, 2003; Seeber and Fastl, 2008; Dorman et al., 2015), and their ITD sensitivity is generally supposed to be poor (Long et al., 2006; van Hoesel et al., 2009; Aronoff et al., 2010; Noel and Eddington, 2013). However, a role of the envelope ITD processing in directional hearing with CI is possible (Todd et al., 2019), since envelope ITD cues are preserved during CI preprocessing.

Speech understanding in noise is aided by at least three effects: first, the head shadow effect, a benefit resulting from listening with the ear with the better signal-to-noise ratio (SNR) compared to not preferring either ear. For instance, if the head is between the signal and noise locations, the ear pointing toward the signal is shielded from the noise by the head, and thus mostly receives the signal. Second, binaural squelch arises by adding an ear with a poorer SNR compared to listening with only the ear with the better SNR. These effects rely on ILD. Thus, ILD not only aid sound localization, but also speech understanding in difficult listening conditions. Third, listening with both ears results in an improved speech intelligibility compared to listening with only one ear, even when there is no spatial separation between the signal and the noise. This is called binaural redundancy.

The present study explores localization ability in individual subjects to differentiate between good and poor performers. Localization ability on the CI and NH sides was measured separately and compared to age-matched controls. This allowed testing the hypothesis that benefits in speech-in-noise understanding mainly appear at locations that also show improved localization with CI. Varying the spatial relation of noise and signal allowed to distinguish improvements due to head shadow and binaural redundancy effects. Since ITD processing is expected to be poor in CI participants, low- and high-pass filtered noises were used to investigate potential differences between the contribution of ITD and ILD to sound localization in CI participants.

Several studies found significant improvement in localization in SSD participants provided with a CI (Dorman et al., 2015; Távora-Vieira et al., 2015; Mertens et al., 2016; Dillon et al., 2017b). Further studies measured localization and additional speech-in-noise understanding (Arndt et al., 2011; Firszt et al., 2012; Gartrell et al., 2014; Grossmann et al., 2016; Buss et al., 2018; Dirks et al., 2019; Litovsky et al., 2019). However, several studies measured the relationship between spatial release from masking and localization abilities, but the aim of the present study was to correlate speech-in-noise understanding for a given location with localization accuracy. This is an important question, because if spatial hearing can be leveraged to significantly improve speech understanding in these patients, then, CI manufacturers are encouraged to consider, e.g., the cues underlying spatial hearing in the development of CIs, and clinicians may pay more attention to localization during rehabilitation. Our study goes beyond previous work in that we avoid visual capture (hidden speakers), measure directional localization errors, relate the patients’ performance to a much larger control group of 129 participants, and differentiate between the CI side and the normal-hearing (NH) side.

## Materials and Methods

### Participants

The study included 18 adults aged 24–81years (mean age: 55.8, SD: 18.2years, 13 females, and five males). All participants were provided with a cochlear implant on one side and had normal hearing on the other side. The participants’ audiometric thresholds on the normal-hearing side were 20dB hearing level or better, at octave frequencies from 250 to 8,000Hz (ANSI, 1996). Ten of them had their CI on the left and eight on the right side. The mean duration of SSD was 2.6years (range: 0.3–8.7); the mean duration of CI usage was 11.3months (range: 1.1–31.5; values for all individuals are listed in Table 1).

**Table 1:** Participants.

ID	Cause of SSD	Duration of SSD (months)	Duration of CI usage (months)
1_51	Sudden hearing loss	48	8.0
2_46	Accident	63	9.7
3_27	Sudden hearing loss	91	1.1
4_59	Sudden hearing loss	69	2.8
5_39	Otitis media	26	14.6
8_65	Sudden hearing loss	24	16.3
9_25	Progressive hearing loss	3	8.7
10_74	Sudden hearing loss	22	5.5
11_55	Sudden hearing loss	22	3.5
12_75	Accident	5	31.5
13_81	Sudden hearing loss	34	12.0
14_77	Sudden hearing loss	9	7.0
15_45	Sudden hearing loss	5	8.5
17_64	Stapes surgery	50	25.3
18_68	Sudden hearing loss	13	7.0
19_24	Unknown		
20_71	Acute hearing loss	15	11.1
21_59	Sudden hearing loss	22	19.2

*Demographic data showing individual ID (testID_age), cause, and duration of single-sided deafness before CI implantation and duration of subsequent CI usage*.

All participants were patients of the clinic of Otorhinolaryngology at the Unfallkrankenhaus Berlin and/or of the hearing therapy center Potsdam. All subjects had been familiar with audiometric testing in general, i.e., speech recognition tests, but were unfamiliar with the test setting of the present study. The participants gave their written informed consent for participation. Clinical speech tests and the experimental localization tests were conducted independently on different days. This study was conducted according to the World Medical Association Declaration of Helsinki and approved by the local Ethics Committee of the University of Leipzig.

### CI Signal Processing

Participants were tested using their standard CI setting. Fifteen participants had a Nucleus implant using an advanced combinational encoder (ACE) strategy and a CP910 Nucleus 5® speech processor from Cochlear™. Three participants had a Sonata implant using FS4 (fine-structure) coding strategy and an Opus 2 speech processor from Medel. The microphones of both processors were set to “omnidirectional.” Both processors transfer the envelope of the auditory signal. Only the Medel processor additionally transfers some of the temporal (low-frequency) fine-structure information of the stimuli from four electrodes.

Auto-sensitivity (ASC, Cochlear®) or Automatic gain control (AGC, Medel) were activated for all participants but did not influence our experimental stimuli, because they activate at higher intensities.

### Setup

**Figure 1:**
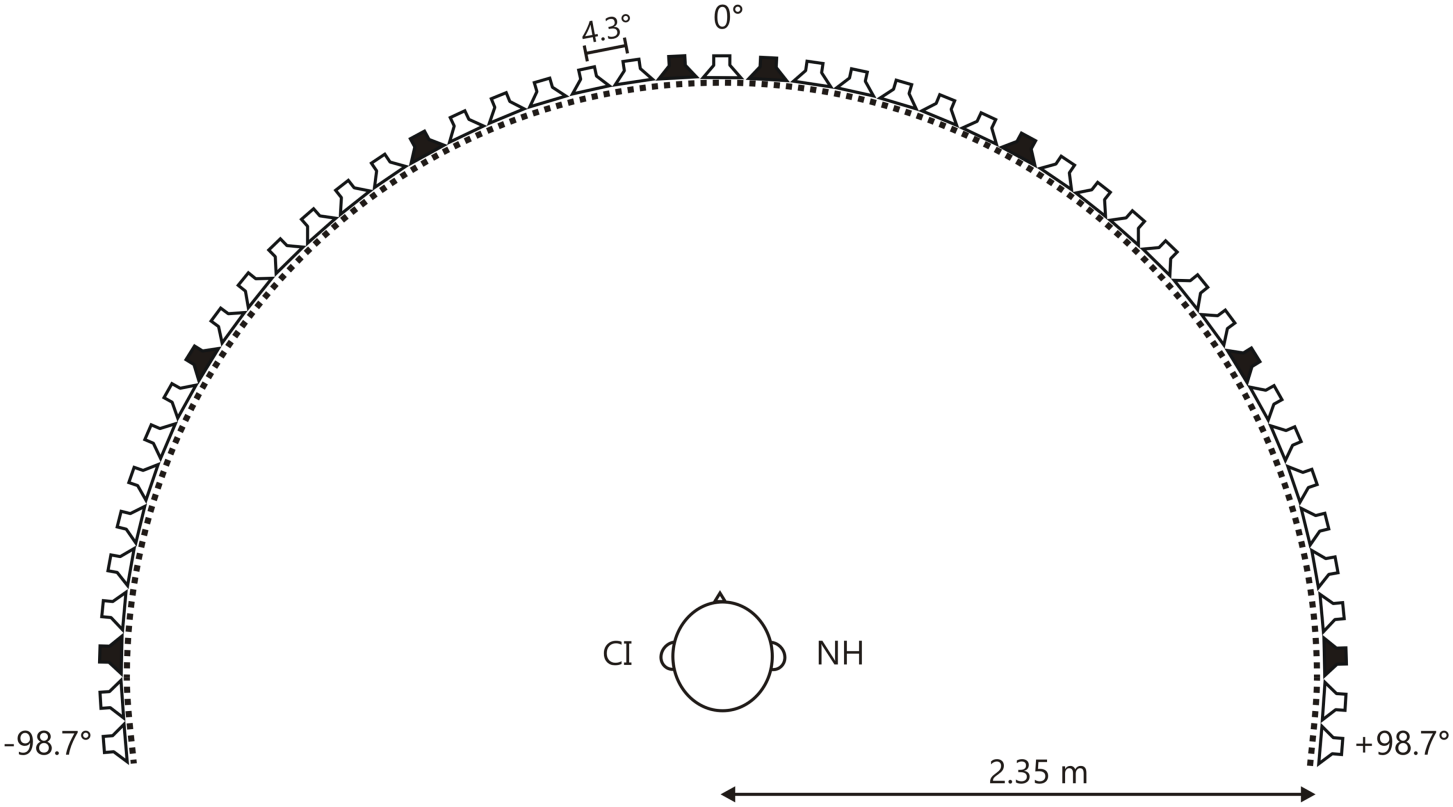
Loudspeaker array. Forty-seven speakers placed on a semicircular array with a radius of 2.35m. The separation between speakers was 4.3°. The head of the participant was directed toward 0°. Test locations were ±4°, ±30°, ±60°, and ±90° (black loudspeakers).

All psychoacoustic testing was performed in a darkened, anechoic, and sound-attenuated room (40m^2^; Industrial Acoustics Company, Niederkrüchten, Germany) free from distracting elements. Forty-seven custom designed loudspeakers (VISATON FRS8) were arranged in a semicircular section (radius 2.35m, with the subject in the center position) spanning the front of the subject from −98° to +98°; the angular separation between the loudspeakers was 4.3° (Figure 1). Each loudspeaker’s transfer function was equalized. For this, the transmission spectrum was measured using a Bruel & Kjaer measuring amplifier (B&K 2610) and microphone (B&K 2669, preamplifier B&K 4190) and a real-time signal processor (RP2.1; Tucker-Davis Technologies, TDT, Alachua, Fla., United States). An inverse filter was computed and later used for generating acoustic stimuli with flat spectra across the stimulus frequency range (300–8,000Hz). This calibration minimized spectral differences between loudspeakers. The entire loudspeaker array was covered by black, acoustic transparent gauze to prevent the participants from seeing the number, the location, and the spatial distribution of potential sound sources. The participants were seated in the center of the loudspeaker array in a comfortable seat equipped with a headrest, with the head oriented to the 0° azimuth indicated by a white LED light spot.

The speaker array was combined with an array of 188 white light emitting diodes (LED, 2.52 lux, 0.6° visual angle) mounted in azimuthal steps of 1° at eye level. The LEDs were controlled by 51 printed circuit boards, which were arranged on top of the loudspeakers. Four infrared (IR) sensitive phototransistors were mounted on each board, arranged at the same angular distances as the LEDs, but covering an additional 8° on both sides. A customized infrared torch served as pointing device (IR-torch, Solarforce L2 with 3W 850nm NVG LED, Fulidat Electronics Limited, Kowloon, Hong Kong). The subtended angle of the IR light beam covered a maximum of 8° at the level of the LEDs. The mean location of all activated IR-sensitive phototransistors was computed online, and the corresponding LED flashed up as a visual feedback of the pointing direction for the participant.

### Speech-in-Noise Task

#### Oldenburger Satztest

Speech understanding in noise was tested with the Oldenburger Satztest (OLSA, Wagener et al., 1999a,b,c,d). The noise was a male two-talker babble noise (Wagener and Brand, 2005) at a fixed level of 65dB SPL (sound pressure level). Thirty sentences of five words (for example: “*Peter kauft fünf grüne Messer*.” – “Peter buys five green knives.”) that started at an SNR of −10dB (speech level=55dB SPL) were presented. The level of the speech was adapted in a 1-up 1-down staircase procedure (Levitt, 1971) to measure the speech reception threshold (SRT), at which 50% of the test material was repeated correctly. Subjects performed this staircase procedure two times for familiarization and training before SRT was measured twice, with and without CI. Speech signals were presented from 45° at the CI side, and noise presented from 45° at the normal-hearing side. The head shadow effect was then determined as the difference between the conditions with and without CI, because adding the CI leads to a better SNR at that side. We chose 45°, because, at this angle, sources are within the visual field and speech and noise sources can be placed symmetrically around the midline with an appropriate separation. The exact angle is likely not too significant; Kühnle et al. (2013) and Ludwig et al. (2019) found no difference between localization accuracy at 30° and 45°. This is a fairly ecologically valid listening situation. Due to the participants’ time constraints, it was not possible to test the OLSA in the S0°/N0° condition.

#### Hochmair-Schulz-Moser

The German Hochmair-Schulz-Moser sentence test (HSM) measures speech understanding in noise. It consists of 30 lists, each with 10 three-to-eight word everyday sentences (“*Ist die Kanne leer*?” – “Is the jug empty?”; Hochmeier-Desoyer et al., 1997), which were presented concurrently with a noise with a speech-shaped spectrum. The noise was presented at 65dB SPL and the speech at an SNR of −10dB (speech level=55dB SPL). SRT were calculated as for the OLSA. Each subject underwent two training sessions before the actual test run. Speech and noise signals were presented at 0° azimuth. The binaural redundancy effect was then determined as the difference between the conditions with and without CI, because binaural redundancy would lead to a benefit when listening with two ears even when there is no difference in SNR. The HSM data were acquired during clinical routine and served as a convenient control, because one would not expect an effect of localization ability for co-located signals.

### Localization Task

#### Stimuli

Stimulus generation and test procedures were controlled by Matlab® (2007b; Mathworks Inc., Natick, Mass., United States). Stimuli were digitally generated by two PC-controlled instruments from TDT (RX8 modules System III) devices (Tucker-Davis Technologies, TDT, Alachua, Fla., United States).

Stimuli were low-frequency (LF, 0.3–1.2kHz) or high-frequency (HF, 2–8kHz) Gaussian noise bursts with Kaiser-filter-shaped envelopes. These spectra were chosen to selectively address binaural signal processing based on ITD or ILD. Both noises had a bandwidth of two octaves. Signal duration was 500ms. Signals were presented at 40dB sensation level (see “Individual Determination of Stimulus Intensity”). The level of the stimuli was not roved, because there seem to be no influence of different levels on localization (Dillon et al., 2017b; Buss et al., 2018).

#### Individual Determination of Stimulus Intensity

Individual hearing thresholds for LF and HF signals were obtained from 0° at the beginning of each testing session using a staircase (heard/not heard) procedure. Starting at a level of 60dB SPL, intensity was decreased or increased in 3dB steps. A single test run was terminated after eight turn points. These respective threshold values for both frequency bands were used to set the presentation level for the subsequent tests at 40dB sensation level. The sensation levels were used in order to ensure comparability with the existing normative data. Presentation levels ranged from 50 to 70dB SPL (mean: 62dB SPL).

#### Localization

Auditory localization was tested for eight azimuthal locations: frontal (±4°), mid-frontal (±30°), mid-lateral (±60°), and lateral (±90°). Each location was tested five times in random order. LF and HF signals were tested separately, resulting in 80 signal presentations (eight locations×five repetitions×two stimulus conditions). The participants were instructed to face the 0° loudspeaker and look at a fixation point during stimulus presentation. After each signal presentation, the participants were asked to indicate the perceived sound location with the infrared torch. For that, they were allowed to turn their head to the perceived sound location, after which they again faced straight forward. Prior to actual testing, participants were presented three practice trials to familiarize themselves with the procedure. This test requires very little cognitive effort and has previously been used for the evaluation of spatial hearing skills in adults with acquired brain lesions (Witte et al., 2012) and schoolchildren (Kühnle et al., 2013). Thus, the test procedure is suitable for use in the present participant group. Depending on the age of the participants, their ability to concentrate, and the individual need for breaks, test sessions took about 2h.

### Statistical Analysis

The median performance from five signal presentations of every SSD participant was quantified as signed angular distance from the direction of pointing and will be referred to as “relative localization.” The “absolute localization” accuracy was quantified as median of the absolute difference between indicated and actual sound source location across the five stimulus presentations. To obtain a single subject analysis independent of the age of the participants, these values were then standardized (*z*-transformed): *z*-score=(*x* - x-_norm_)/std.(x_norm_), using mean and SD of the age-matched normative group. Normative groups comprised cohorts of adults aged 20–29, 30–39, 40–59, and 60–79years. *Z*-scores above 1.64 indicate a significant one-tailed *t*-test result at a 5% type I error rate.

Results of the measurements with and without CI in the speech tests were compared using paired *t*-tests (*p*<0.05).

ANOVA and multiple linear regression analyses were computed for the duration of deafness, the duration of CI usage (Table 1), head shadow effect from the OLSA test, and binaural redundancy effect from the HSM test (see “Clinical Setting”) as dependent variables and each test location with normalized localization accuracy as independent variable.

All analyses were calculated for both frequency bands separately. Value of *p* were Bonferroni corrected.

## Results

### Relative Localization Performance

**Figure 2:**
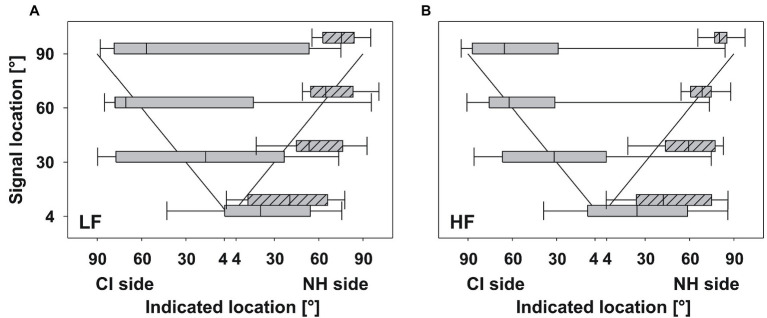
Relative localization. The median across participants of indicated locations plotted against signal locations for **(A)** LF signals and **(B)** HF signals. For consistency across participants, results from the CI side are plotted on the left side of the abscissa, even if the CI was actually on the right side. The diagonal line shows 100% correct localization. Box plots show median (black line), 25th and 75th percentile (boxes), and 10th and 90th percentile (whiskers). Plain boxes indicate localized signals on the CI side, shaded boxes indicate localized signals on the NH side. On average, the group of SSD participants was able to localize the sounds, albeit with high variability.

The difference between the sound locations and the median of the indicated locations was on average 60.6° on the NH side and 43.5° on the CI side (Figure 2). In contrast, mean results from normal-hearing adults varied between 4.1° (SD: 1.9°) and 17.8° (SD: 8.5°; Freigang et al., 2014, 2015).

The variability of the present group was very high. Many of the participants tended to point to the normal-hearing side rather than the CI side. Problems occurred especially for signals from 4° and 90° on the CI side, in particular for LF signals. The interquartile range of the responses varied, e.g., between 129.4°, for LF signals from 90° on the CI side, and 40.7°, for HF signals from 90° on the CI side (Table 2). Performance for HF signals was better than for LF signals.

**Table 2:** Descriptive statistics for localization.

	Auditory localization							
	Signal presentation on the CI side				Signal presentation on the NH side			
	90°	60°	30°	4°	4°	30°	60°	90°
**LF**								
Median	56.7°	70.6°	16.6°	*20.6°	40.4°	53.3°	64.7°	75.4°
25th	77.0°	77.0°	77.0°	3.2°	15.0°	46.0°	54.6°	63.1°
75th	*52.4°	*4.3°	*34.2°	*52.4°	65.3°	72.8°	82.4°	83.5°
**HF**								
Median	65.3°	62.1°	31.6°	*24.6°	42.3°	59.4°	68.5°	80.3°
25th	86.7°	73.3°	65.3°	7.5°	26.8°	43.9°	61.0°	78.1°
75th	46.0°	47.1°	3.2°	*54.6°	73.8°	77.0°	72.8°	84.5°

Median, 25th and 75th percentiles separated for signal presentations on the CI side and on the NH side. Data for LF and HF stimuli, separately. Values preceded by an asterisk (*) were localized on the NH side (contralateral to the sound location).

The majority of participants (*n*=15) was able to differentiate between signals on the CI side and on the NH side for every signal location except 4°. We refer to these participants as “good performers,” because they consistently identified the stimulated side, despite having to integrate acoustic and electrical hearing. The remaining three participants only pointed to the NH side irrespective of the signal location (“poor performers”).

#### Good Performers (*n*=15)

**Figure 3:**
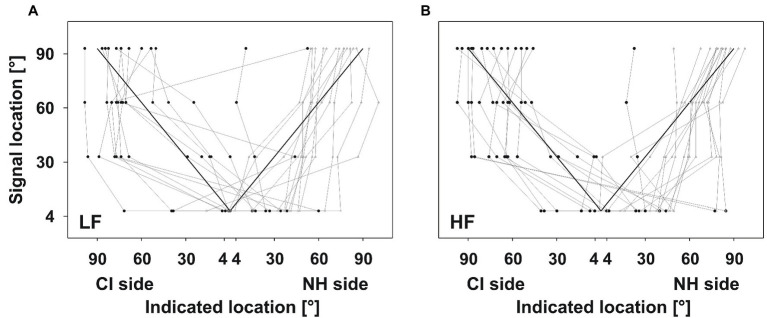
Good performers. Indicated locations plotted against the signal location. Layout of the plots as in Figure 2. Symbols show the median of five signal presentations for every participant, separately. Black circles indicate signals presented on the CI side, whereas gray triangles indicate signals presented on the NH side. Participants were able to identify signals on the CI and on the NH side. Some of them showed a trend for correct localization from frontal to lateral signal locations.

Although the variability of the pointing behavior was quite large, all good performers were able to identify signals on the CI and on the NH side (Figure 3). Some of the participants showed a trend for correct localization from frontal to lateral signal locations. Signals close to the midline (4°) on the NH side were localized to the correct side. Only signals close to the midline (4°) on the CI side were mislocalized to the NH side by about half of the participants.

When comparing the two frequency bands, performance in the HF condition tended to be closer to the correct localization than in the LF condition. One participant was able to localize LF signals but not HF signals. Two participants were able to localize HF signals but not LF signals.

#### Poor Performers (*n*=3)

**Figure 4:**
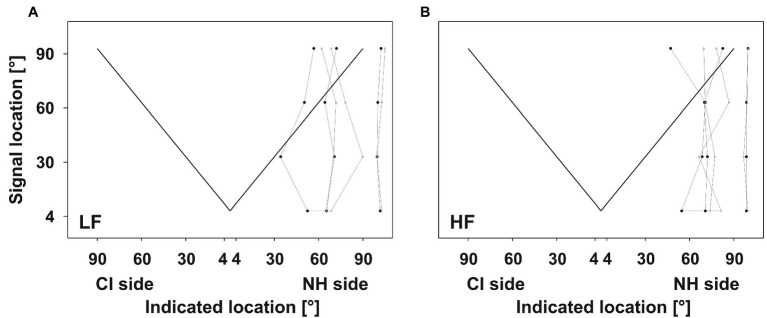
Poor performers. Layout of the plots as in Figure 3. Participants did not benefit from their CI for localization; all responses indicated the NH side irrespective of signal location.

Three participants did not seem to benefit from their CI regarding localization for both frequency bands (Figure 4). Participants localized all signals on the NH side, regardless of whether signals were presented on the CI side or on the NH side. Indicated locations ranged from 20° to 90° or even further lateral to the end of the loudspeaker array. Participants did not show a trend of increasingly lateral responses to increasingly lateral signal locations, comparable to what was found in good performers (see Table 2).

#### Performance Without CI

**Figure 5:**
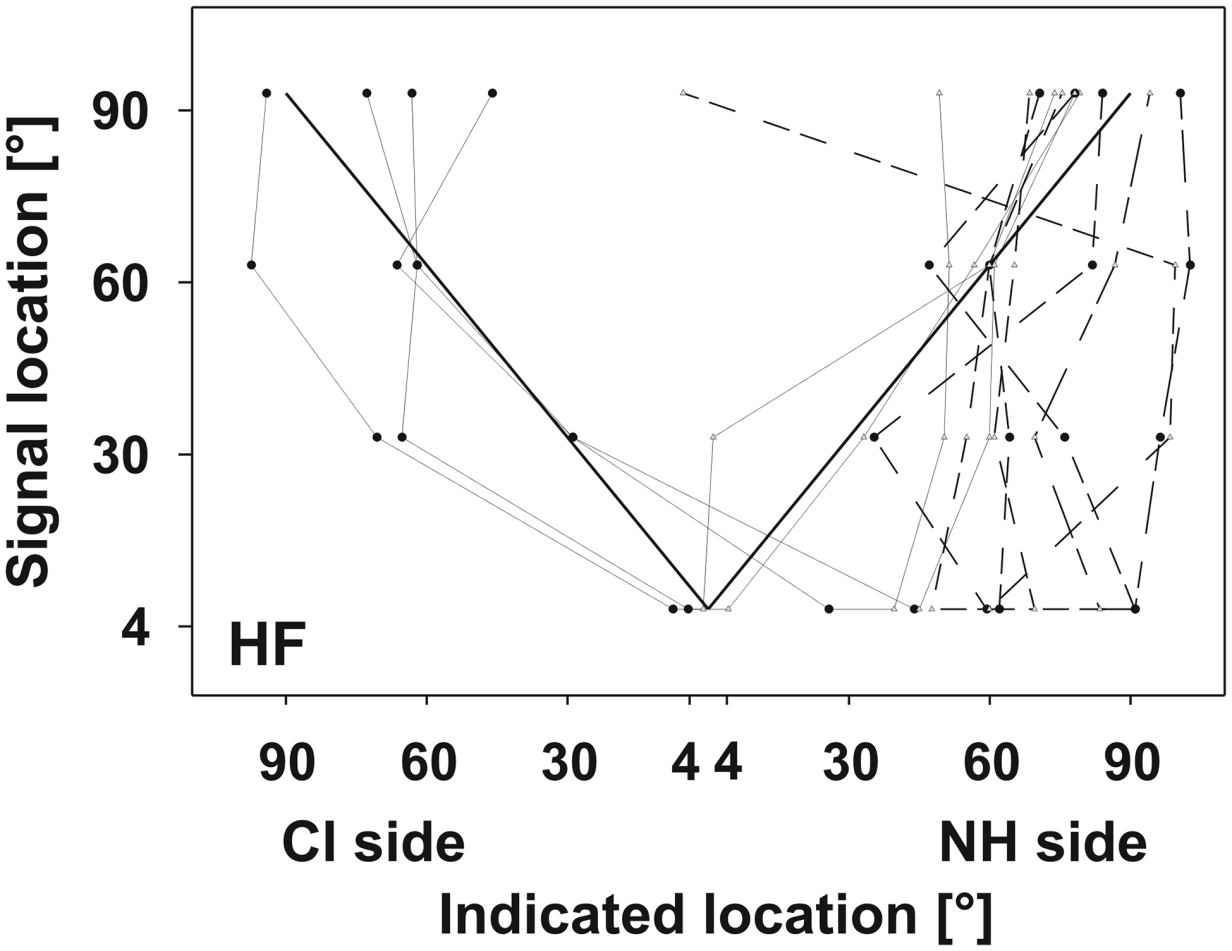
Monaural vs. binaural test for HF signals. Layout of the plot as in Figure 3. Solid lines indicate performance with the CI (identical to Figure 3). Dashed lines indicate performance without the CI, which was comparable to the poor performers.

Four of the good performers were also tested without the CI. HF signals were presented, because these had yielded better localization performance than LF signals. As expected, the participants could not differentiate the stimulated side and indicated the NH side irrespective of signal location, comparable to the poor performing participants (Figure 5). Indicated locations mostly ranged between 45° and 90° on the NH side.

### Absolute Localization Performance

Age is a main factor in explaining changes in localization performance (Freigang et al., 2015). Thus, we compared absolute localization to age-matched control data (Freigang et al., 2014; Table 3).

**Table 3:** Normative data.

Signal location	20–29Years, *n*=22	30–39Years, *n*=23	40–59Years, *n*=20	65–83Years, *n*=64
	Mean (SD)	Mean (SD)	Mean (SD)	Mean (SD)
LF −90°	9.3° (4.6°)	6.9° (4.4°)	10.0° (6.5°)	9.5° (6.9°)
LF −60°	7.4° (4.8°)	5.4° (3.2°)	7.1° (4.1°)	7.7° (6.5°)
LF −30°	4.5° (3.9°)	3.7° (2.4°)	4.8° (2.6°)	6.0° (6.0°)
LF −4°	3.0° (2.1°)	2.3° (1.5°)	3.4° (1.6°)	3.8° (3.8°)
LF 4°	3.6° (2.8°)	3.0° (1.0°)	3.1° (1.7°)	3.2° (2.8°)
LF 30°	3.3° (2.3°)	2.8° (1.1°)	4.1° (3.7°)	2.8° (2.8°)
LF 60°	6.2° (4.8°)	4.4° (2.6°)	6.8° (4.6°)	6.6° (5.8°)
LF 90°	8.0° (4.6°)	4.9° (3.3°)	7.8° (5.9°)	10.3° (8.0°)
HF −90°	11.1 (6.2)	11.6 (7.5)	14.4 (16.8)	15.3 (9.7)
HF −60°	5.1 (3.6)	5.7 (3.8)	12.4 (7.5)	10.0 (8.5)
HF −30°	3.7 (3.2)	3.2 (1.9)	6.8 (4.9)	7.8 (12.8)
HF −4°	3.1 (2.6)	2.8 (1.9)	3.4 (1.5)	5.6 (10.1)
HF 4°	3.1 (3.1)	2.9 (1.8)	3.4 (2.6)	4.2 (3.6)
HF 30°	4.9 (5.6)	3.0 (2.1)	7.6 (9.9)	4.8 (5.4)
HF 60°	5.2 (4.3)	5.3 (2.6)	10.2 (9.0)	9.5 (7.2)
HF 90°	9.9 (7.2)	8.6 (8.3)	10.4 (9.1)	13.5 (7.9)

*Results for absolute localization in normal-hearing adults for each location, separately. – indicating the left side and+indicating the right side of the participants. Data for four cohorts and the respective number of NH participants as mean and SD*.

**Figure 6:**
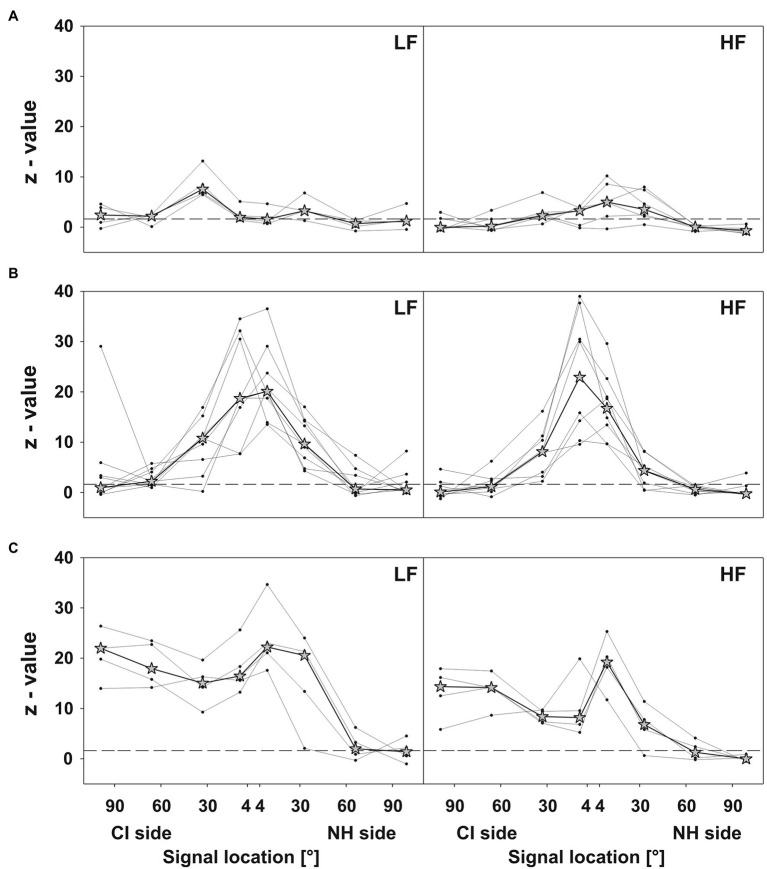
Absolute localization. *Z*-values plotted against the signal location for LF (left panels) and HF (right panels) signals. The dashed lines depict the significance level (1.64). Dots depict the *z*-value compared to the actual sound source location for every participant, separately. Asterisks depict the respective median of the group. Three different response patterns were evident: **(A)** good performance (at least four *z*-values below or nearby 1.64), **(B)** reduced accuracy at frontal locations, and **(C)** reduced accuracy on the CI side.

A few (four for LF signals and six for HF signals) of the good performers from the relative localization test also showed good performance in the absolute test (Figure 6A). Although z-values increased up to 13.1 at some positions, most were below 1.64.

The remaining good performers (nine for LF signals and eight for HF signals) predominantly showed reduced accuracy at frontal positions (Figure 6B), with the best localization at 60° and 90° on both sides. At 30° and 4°, these participants performed 4–40 SDs (*z*-value) above (i.e., worse) the average localization accuracy of age-matched controls.

All poor performers had reduced localization accuracy at all positions, except 60° and 90° on the NH side (Figure 6C). Although *z*-values for HF signals were lower than for LF signals, they reached up to 25 on the NH side.

### Speech-in-Noise Tests

Speech-in-noise tests were conducted with and without the CI. Differences in speech-in-noise reception thresholds (SRT) between both conditions describe the head shadow effect for the OLSA test and the binaural redundancy effect for the HSM test (Table 4).

**Table 4:** Speech test results.

ID	OLSA SSD	OLSA CI-NH	Difference OLSA dB	HSM SSD	HSM CI-NH	Difference HSM dB
1_51	−3,4	−5,5	−2,1	−3,75	−3,7	0,05
2_46	−4,9	−6,1	−1,2	−3,25	−4,6	−1,35
3_27	−1,1	−4,1	−3	−3,5	−3,2	0,3
4_59	3,75	0,55	−3,2	−4,55	−5,4	−0,85
5_39	1	−2,9	−3,9	−4,4	−3,6	0,8
8_65	−2,25	1	3,25	−3,3	−2,25	1,05
9_25	4,3	0,6	−3,7	−2,8	−3,3	−0,5
10_74	0,7	0,9	0,2	−2,1	−3,25	−1,15
11_55	1,7	−5,3	−7	−5,2	−5,1	0,1
12_75	1,4	−1,9	−3,3	−5,6	−5,6	0
13_81	0	−0,6	−0,6	−1,9	−3,4	−1,5
14_77	1,5	0,4	−1,1	−3,1	−3,3	−0,2
15_45	−1,5	−5,4	−3,9	−5	−5,1	−0,1
17_64	−0,3	−3,3	−3	−6	−6,3	−0,3
18_68	1,8	0	−1,8	−3,6	−3,5	0,1
19_24	2,3	0,3	−2	−5	−4,4	0,6
20_71	−0,1	0	0,1			
21_59	−2,9	−4,3	−1,4	−0,9	−2,6	−1,7
Paired *t*-test			*p* <0.001			*p* =0.18

*Values show SRT determined without CI (SSD, columns 2 and 5) and with CI (CI-NH, columns 3 and 6) and the difference between both conditions (columns 4 and 7). The differences represent the head shadow effect for the OLSA test and the binaural redundancy effect for the HSM test. Lower values in columns 4 and 7 denote the effect of adding the CI to the SSD condition*.

Fifteen participants performed better in the OLSA test with their CI than without it: SRT values improved by 0.6 to 7dB (mean: −2.75dB). This improvement was statistically significant [t_(17)_=−4.072, *p*<0.001]. Three participants showed no improvements or performed slightly worse with CI. Interestingly, those participants also showed poor relative localization performance (poor performers; Figure 4) and had most problems on the CI side in the absolute localization performance (Figure 6C). No difference between with and without CI conditions was found in the HSM test.

### Regression Analysis

An ANOVA with a multiple regression model was computed concerning the influence of localization ability at different directions on the head shadow effect (OLSA test), and the influence of the duration of deafness and the duration of CI use on the localization ability. The binaural redundancy effect was not included in this analysis, because no difference between conditions was found.

**Figure 7:**
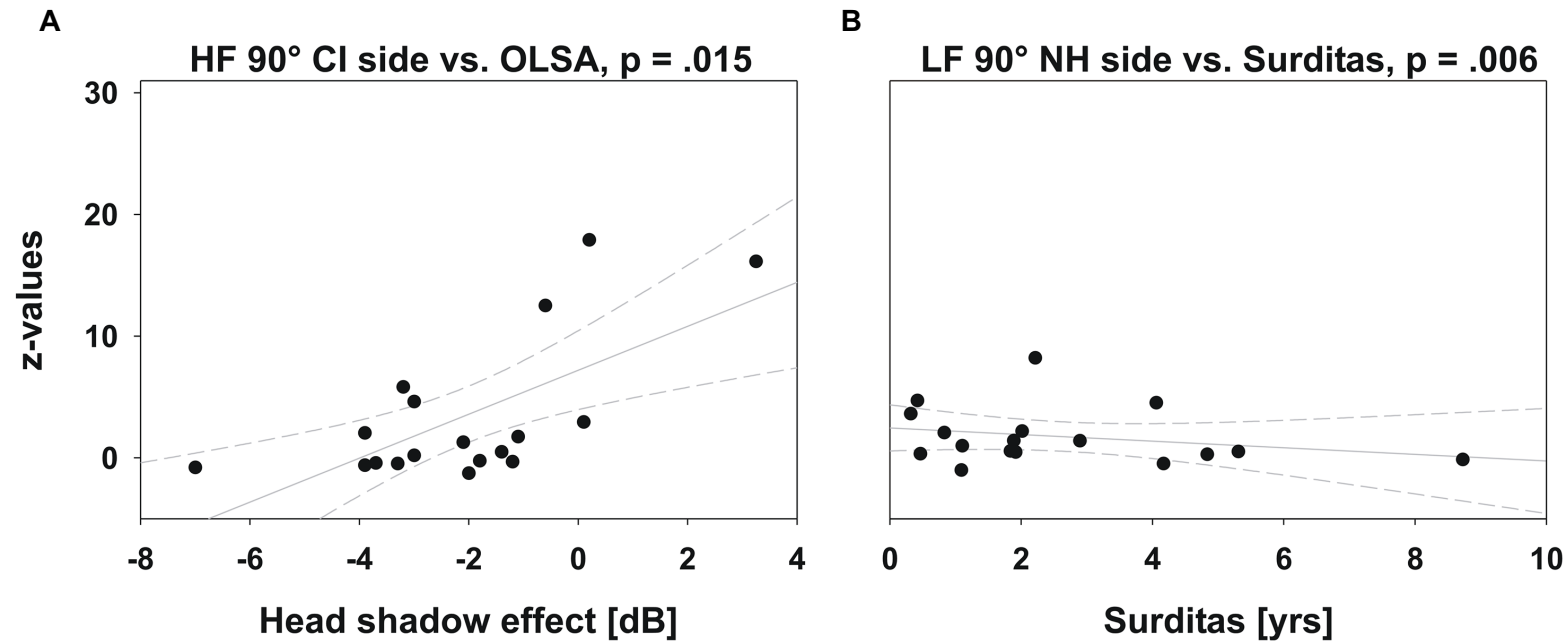
Multiple regression. Localization ability (*z*-values) plotted against head shadow effect (OLSA test) and duration of deafness. The gray solid line depicts the regression line and 95% CI (short-dashed lines). **(A)** The regression between OLSA test performance and localization ability was only significant at 90° on the CI side, where speech understanding in noise was also improved by the CI. Participants who localized sounds more accurately (lower *z*-scores) showed greater improvement in SRT due to the CI. **(B)** Although the ANOVA revealed a significant influence of the duration of deafness on the localization ability, the coefficients are not clinically relevant, as shown here for the regression with the lowest value of *p*.

The benefit of the CI in the OLSA test was related to absolute localization ability across directions for HF signals [*F*_(8,17)_=3.228, *p*=0.05]. *Post hoc T*-tests revealed that more accurate localization at 90° on the CI side implied a larger benefit of the CI in speech understanding, as measured by the OLSA test [*t*_(18)_=2.99, *p*=0.015, Figure 7A]. The correlation at 30° and 60° on the CI side showed a strong trend (*p*=0.054 and *p*=0.058, respectively). The regression appears to be mainly driven by participants who were unable to localize sounds on the CI side, and none of which benefitted from the CI in this test.

The influence of the duration of deafness on the localization ability was statistically significant for LF signals [*F*_(8,16)_=3.492, *p*=0.048], but the coefficients of the resulting model were very small and would be clinically meaningless (Figure 7B). This correlation was not significant for HF signals; neither was the influence of the duration of CI use on the localization ability.

## Discussion

To our knowledge, this is the first study, which analyzed the localization accuracy on the CI side and the NH side, separately. Absolute localization with respect to normative data and relative localization with respect to the direction of error was evaluated. Localization accuracy was correlated to speech-in-noise understanding at different locations.

The majority of participants (15 out of 18) was able to localize sounds coming from the CI side and to differentiate between sounds from the left and the right hemi-field. Results from these good performers showed that wearing a CI can restore localization for signals coming from the CI side and slightly enhance localization of signals on the NH side. Individual results showed that about a quarter of the participants demonstrated localization abilities close to those of normal-hearing controls, about half of the participants localized less accurately at frontal locations, and another quarter could localize sounds at the NH side only. These performance differences have not been reported before, because previous work focused on summary measures of accuracy and did not differentiate between NH and CI side. Participants who could not localize sounds on the CI side also did not benefit from the CI in the speech-in-noise understanding.

### CI-SSD Participants Were Able to Use ITD Cues for Localization

Recent studies have found that SSD participants provided with a CI (Firszt et al., 2012; Dorman et al., 2015; Mertens et al., 2016; Dirks et al., 2019) as well as bilateral CI users (Long et al., 2006; Seeber and Fastl, 2008; van Hoesel et al., 2009; Aronoff et al., 2010; Noel and Eddington, 2013) can localize high-frequency signals better than low-frequency signals. This is probably because most CIs code the sound envelope, i.e., temporal changes in amplitude, but not temporal fine-structure, the basis for ITD (Wilson and Dorman, 2009). Thus, Dirks et al. (2019) suggested that CI-NH listeners were unable to use ITD cues. Dorman et al. (2015) showed that CI-NH listeners primarily rely on ILD, although their participants were able to localize low-pass noise with above-chance accuracy. Almost all of our participants localized HF stimuli more accurately than LF stimuli, but 13 participants correctly identified the hemi-field even in the LF condition. Because there were no appreciable ILD in this condition, 87% of our SSD participants with a CI appeared able to use ITD information.

The main information carrier for sound localization is temporal fine-structure ITD (Kistler and Wightman, 1992; Smith et al., 2002). Three participants were fitted with CIs from Medel, using a processing strategy that preserves some temporal fine-structure. One of them derived no measurable localization benefit from the CI for LF and HF signals. The other two correctly identified the hemi-field in both conditions. All other participants were fitted with CIs from Cochlear, not using a fine-structure preserving processing strategy. However, 12 of them showed good performance for LF and HF signals. Dirks et al. (2019) found no differences between different processing strategies (Medel and Advanced Bionics) although their participants only relied on ILD. They argued that temporal fine-structure does not contribute to localization in CI participants. However, synchronized delays between both ears may be necessary for effective fine-structure cues, but this is not possible with a normal-hearing ear and a CI and would require binaural aided hearing.

Three participants that underperformed in localization never pointed to the CI side, so an imbalance in hearing thresholds between the CI and the NH side could have had an influence on the performance. At the beginning of each testing session, individual hearing thresholds for LF and HF signals were obtained from 0°. The sensation level was set with respect to the CI side, assuming that, after one to 31months of CI use, the CI and the NH side should have achieved comparable hearing levels (Keating and King, 2013). Signals were presented 40dB above this threshold in the localization test. In CIs, signal level information is severely compressed by an automatic gain control. For instance, after CI preprocessing, the ILD of a 3kHz tone at 15° is 0.4dB (3dB in NH), and at 45° it is 1.6dB (10dB in NH, Dorman et al., 2014). Thus, SSD-CI participants experienced compressed ILD. Envelope ITD cues, although linearly offset by the processing delay of the CI, were in principle available to all participants, but the three poor performers were apparently unable to use them. Note, however, that envelope ITD cues are strongly reduced in CI patients due to spectral smearing (Oxenham and Kreft, 2014). Another possibility is that these participants did not adapt to the constant intensity difference between the normal ear and the CI.

### Relation Between Localization Performance and Duration of Deafness and Experience With CI

Four of the good performers were tested twice, binaurally with the CI (CI-NH condition) and without wearing the CI (SSD condition) and showed a demonstrable benefit in the CI-NH condition. These results show that a CI can restore localization ability. This is in accordance with several studies who showed that performance was significantly better in the CI-NH condition compared to the SSD condition (localization and speech-in-noise test: Firszt et al., 2012; Gartrell et al., 2014; Grossmann et al., 2016; localization: Mertens et al., 2016; Litovsky et al., 2019).

Some researchers argued that the localization deficits in SSD participants might be related to the duration of deafness, but the results are inconsistent. Távora-Vieira et al. (2015) found no difference in localization accuracy in CI participants who were deaf for less than 10years and those who were deaf for longer than 10years. However, Wedekind et al. (2020), using the same setup as Távora-Vieira et al. (2015), found a correlation such that CI participants with shorter durations of deafness showed greater improvement in localization ability. Buss et al. (2018) argued that there might be a relation between improved localization ability and a reduced side-bias in participants with a short duration of deafness. However, participants with uncompensated (no CI) unilateral hearing loss appear to improve in localization accuracy over time. Firszt et al. (2017) measured pre- or perilingually deafened participants (mean age: 25–71years) with a mean duration of deafness of 21.9years. These participants had a better localization performance than participants with normal-hearing listening unilaterally. Furthermore, the authors found better localization in participants with longer lasting deafness (25–72years of SSD) compared to recently deafened participants (duration <1–3years of SSD). Liu et al. (2018) reported similar results. Slattery and Middlebrooks (1994) measured SSD participants with a duration of deafness of at least 20years and found a shift of responses to the side of the normal-hearing ear in SSD participants. However, they also found SSD participants that could differentiate between the SSD and the NH side. Thus, monaural information (such as monaural spectral cues) is useful for localization, and participants appear to develop their ability to use this information with time (Keating and King, 2013). In our data from participants with a mean duration of deafness of 2.6years, there was no correlation between the duration of deafness and localization accuracy, which confirms the results of Wedekind et al. (2020) and Buss et al. (2018).

We found no correlation between duration of CI usage and absolute localization accuracy. Gartrell et al. (2014) and Wedekind et al. (2020) also found no correlation between different durations of CI usage and localization ability. Dillon et al. (2017b), and Buss et al. (2018) showed that benefits consistently appear at 1month after implantation and increase up to 3months, but there was no further improvement in localization thereafter. Bilaterally implanted CI users (Grantham et al., 2007) showed an improvement after 10months, but only because their first test result was poor. In sum, the present results agree with other recent findings of little or no improvement of localization ability over the duration of CI usage.

### Relation of Localization Accuracy and Speech-in-Noise Understanding

There is inconsistent empirical support for better speech-in-noise perception resulting from a CI in SSD participants: data from Gartrell et al. (2014) showed that the greatest benefit from spatial separation of a target presented from 0° occurred when the masker was located on the side of the implanted ear. Buss et al. (2018) showed a benefit where target and noise were co-located at 0°, whereas Arndt et al. (2011) and Grossmann et al. (2016) showed no such improvement. Our results from the HSM test support the latter two studies, in the case of Arndt and colleagues as a direct replication.

Wedekind et al. (2020) found a significant improvement in speech-in-noise perception regardless of the location of the speech and noise signal. Several studies (Arndt et al., 2011; Grossmann et al., 2016; Mertens et al., 2017; Buss et al., 2018; Dirks et al., 2019) showed a benefit when noise was presented from the NH side. These findings are in accordance with our present results (in the case of Arndt and colleagues again as a direct replication), in which participants showed an improvement in speech understanding in noise, when speech signals were presented from the CI side and noise was presented from the normal-hearing side. Different authors have used different signal configurations to measure the head shadow effect, which limits comparability. In addition, the effect is typically measured as difference between monaural and binaural conditions, and may be contaminated by binaural mechanisms that become available in the binaural condition, such as stream segregation with a subsequent attentional focus on the target steam. Our measurement slightly overestimated the head shadow effect, because the speech source is on the contralateral side in the monaural condition, thus softer than the noise. This results in a slightly negative SNR, compared to a monaural condition with co-located signals (SNR=0). However, this effect significantly correlated with the localization performance at 90° on the CI side. Thus, localization ability and speech understanding in noise might be directly related, when speech and noise are spatially separated and speech is presented on the side on which signals are localized more accurately.

Further studies are needed to investigate, if, e.g., level differences between the CI side and the NH side might impact localization performance. In the present study, the sensation level was set with respect to the CI side. Although Dillon et al. (2017b) found no level-dependent improvement for localization in different conditions, level roving might affect the performance, in that it decreases the use of level cues. Another question is whether differences in CI signal processing would influence the performance. We used processors of two different companies, which differ in (a) processing strategies, (b) stimulation rates, (c) strategies to activate electrodes, and (d) depth of electrode insertion. Minimizing differences in the settings would have helped to understand the differences in performance across participants. Furthermore, additional spatial configurations of the speech and noise signals in the speech-in-noise tests would help to investigate the correlations between localization and speech-in-noise performance with regard to the head shadow effect, binaural squelch, and binaural redundancy.

## Conclusion

Our results highlight the potential way in which individuals with SSD may benefit from cochlear implantation in the deaf ear. It is important to point out that most of the participants regained the localization ability very well or could at least differentiate between signals from left and right, showing that binaural hearing mechanisms had recovered. Furthermore, most localization accuracy was often poor at frontal locations, but this fact might be compensated by vision and head movement in real listening situations. Localization was not only possible with high-frequency signals (mostly ILD cues) but also with low-frequency signals (mostly ITD cues), although the performance was poorer. Participants showed a significant improvement in understanding speech-in-noise in at least one of the speech tests, even if the signals came from the CI side and the noise from the NH side. In essence, these findings provide evidence that the additional auditory input after cochlear implantation in SSD participants enables some binaural hearing mechanisms.

## Data Availability Statement

The original contributions presented in the study are included in the article/supplementary material; further inquiries can be directed to the corresponding author.

## Ethics Statement

The studies involving human participants were reviewed and approved by Ethics Committee of the University of Leipzig; Geschäftsstelle der Ethik-Kommission an der Medizinischen Fakultät der Universität Leipzig c/o Zentrale Poststelle Liebigstraße 18 04103 Leipzig. The patients/participants provided their written informed consent to participate in this study.

## Author Contributions

AL, R-DB, and AE designed the study. AL and R-DB conducted the experiments. AL, SM, and MF analyzed the results. AL and MS wrote the paper. All authors contributed to the article and approved the submitted version.

## Funding

We acknowledge support from the Leipzig University for Open Access Publishing.

## Conflict of Interest

The authors declare that the research was conducted in the absence of any commercial or financial relationships that could be construed as a potential conflict of interest.

## Publisher’s Note

All claims expressed in this article are solely those of the authors and do not necessarily represent those of their affiliated organizations, or those of the publisher, the editors and the reviewers. Any product that may be evaluated in this article, or claim that may be made by its manufacturer, is not guaranteed or endorsed by the publisher.

## Abbreviations

ANOVA: Analysis of variances
CI: Cochlear implant
HF: High frequency
HSM: Hochmaier-Schulz-Moser Test
ILD: Interaural level differences
ITD: Interaural time differences
LF: Low frequency
NH: Normal hearing
OLSA: Oldenburger Satztest
SD: Standard deviation
SNR: Signal-to-noise ratio
SRT: Speech reception threshold
SSD: Single-sided deafness
